# Supplementary material for: Identification of photoperiod-induced specific miRNAs in the adrenal glands of Sunite sheep (Ovis aries)
Source: Front Vet Sci. 2022 Jul 22;9:888207. doi: 10.3389/fvets.2022.888207 (PMC9354845; doi:10.3389/fvets.2022.888207)
Supplement: Supplementary file 1 [file Data_Sheet_1.ZIP › Supplementary file/miRDeep2 file.zip!/miRDeep2 file/Know_Structure/SPLP42A2/oar-mir-17.pdf]

miRBase precursor : oar-mir-17
Total read count : 4080
oar-miR-17-5p read count : 4011
remaining reads : 69

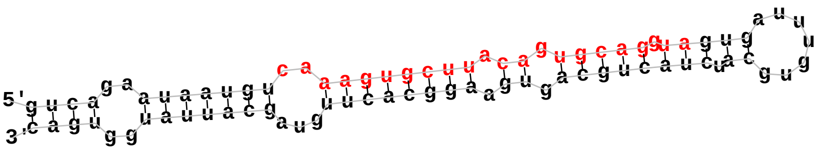

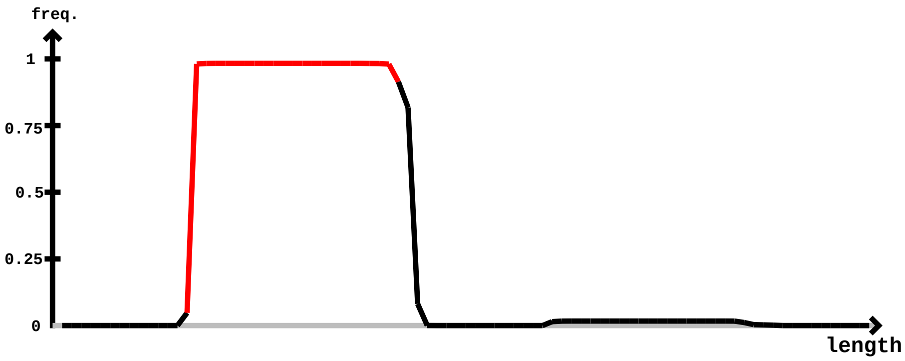


```
                oar-miR-17-5p
5'- gucagaauaaugucaaagugcuuacagugcagguagugauuugugcaucuacugcagugaaggcacuuguagcauuauggugac  -3'    exp
    ((((..(((((((..((((((((.((.(((((.(((((.......)).))))))))).)).))))))))...)))))))..))))   reads     mm     sample
    ...........ucaaagugcuuacagugca......................................................   1         0      seq
    ...........ucaaagugcuuacagugcag.....................................................   1         0      seq
    ...........ucaaagugcuuacagugcagg....................................................   2         0      seq
    ...........ucaaagugcuuacagugcaggu...................................................   23        0      seq
    ...........ucaaagugcuuacagugcaggua..................................................   45        0      seq
    ...........ucaaagugcuuacagugcagguag.................................................   118       0      seq
    ...........ucaaagugcuuacagugcagguagu................................................   5         0      seq
    ............caaagugcuuacagugcagg....................................................   6         0      seq
    ............caaagugcuCacagugcaggu...................................................   3         1      seq
    ............caaagugcuuacagugcaggu...................................................   245       0      seq
    ............caaagugcuuacagugcaggua..................................................   347       0      seq
    ............caaagugcuCacagugcagguag.................................................   37        1      seq
    ............caaagugcuuacagugcagguag.................................................   2843      0      seq
    ............caaagugcuCacagugcagguagu................................................   2         1      seq
    ............caaagugcuuacagugcagguagu................................................   324       0      seq
    ............caaagugcuuacagugcagguagug...............................................   1         0      seq
    .............aaagugcuuacagugcaggu...................................................   1         0      seq
    .............aaagugcuuacagugcaggua..................................................   1         0      seq
    .............aaagugcuuacagugcagguag.................................................   5         0      seq
    ..............aagugcuuacagugcagguag.................................................   1         0      seq
    ..................................................acugcagugaaggcacuug...............   1         0      seq
    ..................................................acugcagugaaggcacuugu..............   22        0      seq
    ..................................................acugcagugaaggcacuugua.............   31        0      seq
    ..................................................acugcagugaaggcacuuguag............   4         0      seq
    ..................................................acugcagugaaggcacuuguagca..........   2         0      seq
    ...................................................cugcagugaaggcacuugua.............   1         0      seq
    ...................................................cugcagugaaggcacuuguagc...........   3         0      seq
    ...................................................cugcagugaaggcacuuguagca..........   4         0      seq
    ....................................................ugcagugaaggcacuuguagc...........   1         0      seq
```
